# Harvestable tumour spheroids initiated in a gelatin-carboxymethyl cellulose hydrogel for cancer targeting and imaging with fluorescent gold nanoclusters

**DOI:** 10.1007/s44164-022-00033-w

**Published:** 2022-10-21

**Authors:** Ashkan Kamali Dashtarzheneh, Amir Afrashtehpour, Bala Subramaniyam Ramesh, Marilena Loizidou

**Affiliations:** Department of Surgical Biotechnology, Division of Surgery and Interventional Science, University College London, Royal Free Campus, Rowland Hill Street, London, UK

**Keywords:** 3D in vitro cancer model, Cancer targeting, Gold nanoclusters, Calreticulin, Gelatin, Carboxymethyl cellulose, Colorectal cancer spheroids, Breast cancer spheroids

## Abstract

Cancer cell spheroids are the simplest 3D in vitro cancer models and have been extensively used for cancer research. More recently, models have been becoming complex, with the introduction of a matrix and non-cancer cell types to mimic specific tumour aspects. However, applying drugs or agents in matrix-embedded cancer spheroids can be problematic. Most matrices can impede and also bind drugs or visualizing agents non-specifically, in the vicinity of the embedded spheroids. This may interfere with imaging or further analysis without breaking apart the 3D model into its constituents. Here, we developed a combined gelatin-carboxymethyl cellulose (G-CMC) hydrogel for initiating cancer spheroids that enabled intact harvesting pre/post treatment for further investigation, such as targeting and imaging. We combined CMC (1.25%) and gelatin (2.5%) at 25 °C and initiated polymerisation after autoclaving (121 °C) to obtain a mechanical strength (sheer stress) of 38 Pas versus 1.28 Pas for CMC alone. These matrix conditions facilitated separation of the spheroids from the G-CMC, using low centrifugation (100 g). We described growth of colorectal and breast cancer spheroids within the G-CMC matrix (with average diameters of 220 mm and 180 μm for representative cell lines HT29 and MCF7 at 10 days, respectively). As the cancer cells express the surface biomarker calreticulin (CRT), we manufactured anti-calreticulin IgG (anti-CRT) conjugated to fluorescent gold nanoclusters (anti-CRT-AuNC) as a probe. We harvested cancer spheroids and incubated live with the nanoclusters. Imaging demonstrated strong binding of CRT-targeted AuNCs compared to control AuNCs. This novel model preserves cancer spheroid integrity upon isolation and is well suited for targeted imaging and drug delivery of cancer in 3D.

## Introduction

Three-dimensional (3D) culture environments facilitate the study of cellular responses in a setting that mimics aspects of the in vivo milieu [1]. Cells in 3D adopt different phenotypic behaviour and expression profiles in contrast to 2D cultured cells. Although the latter offers a “clean” system amenable to delineation of intracellular pathways and mechanisms, it fails to recapitulate the in vivo microenvironment. In contrast, 3D cultures offer the key elements of 3D geometry, which modulate delivery of both nutrients and drug to target cells [2–4], and tissue complexity, i.e., multicellularity and the presence of extracellular matrix proteins and related cross talks, that influence cell behaviour, such as growth and migration, and sensitivity to drugs [5–7]. For this reason, drug evaluation in vitro is increasingly carried out in sophisticated 3D models, away from 2D cultures. However, drug testing and imaging in matrix embedded cancer spheroids can be problematic. The matrix can bind agents non-specifically. Also, spheroids, while embedded, may be difficult to image or further analysed without breaking apart the 3D model. Spheroid isolation from 3D matrices is challenging due to disintegration of the spheroids while separating.

Materials of biological origin and synthetic matrices have been developed to support 3D cell growth, including hydrogels of various compositions. However, harvesting of spheroids from such matrices has not been widely explored. Gelatin is a natural product derived from denaturing type 1 collagen from mammalian [8, 9] and non-mammalian [10] sources. Gelatin preserves natural cell-binding motifs and degradation sites suitable for cell cultivation. It is rich in domains that bind to cell-surface receptors and ECM proteins providing substrates for cell attachment [11]. Depending on the proposed end application, desirable cross-linking agents can be selected. Carboxymethyl cellulose (CMC) is a linear polymeric derivative of cellulose, composed of β-linked glucopyranose residues with partial hydroxyl groups substituted with carboxylmethyl groups. It is water-soluble and forms a viscous solution depending on concentration, and it is widely used as a binder or thickener in pharmaceuticals, foods, and ceramics [12, 13]. When mixed with gelatin while heating, a highly cross-linked hydrogel can be obtained for 3D culture initiation, e.g., for vascular networks [14], and therefore, we hypothesized that it would support harvestable 3D cancer spheroids.

Targeting cancer by drugs or imaging agents can been achieved using nanoparticles conjugated to ligands against biomarkers expressed on cancer cell surfaces. We and others [15] have focused on nanoparticles which can be localized and imaged, to facilitate simultaneous drug delivery and the ability to track. We developed gold-based nanoclusters (AuNCs) which emit in the near infrared (NIR). The mercaptosuccinic acid (MSA)-coated nanoclusters are typically very small (up to 10 nm), with functional groups for conjugation purposes. Nanoparticle size dictates the ability to cross through barriers and into target cells. We also conjugated to an antibody we previously raised against the N-terminal of the calreticulin protein, a biomarker expressed by colorectal and breast cancer cells, to produce targeted NCs (anti-CRT-AuNCs), and demonstrated their binding to cancer cell lines in 2D [16].

In this work, firstly, we developed a 3D cancer model in a gelatin-carboxymethyl cellulose matrix (G-CMC) and demonstrated that colorectal and breast cancer spheroids grow well over time and are harvestable with minimum disruption. Secondly, we incubated harvested spheroids with NCs, to demonstrate uptake by live cancer cells in 3D, and showed much higher binding of CRT-targeted compared to untargeted NCs, as expected. Overall, we describe a versatile 3D cancer model suitable for nanoparticle imaging.

## Materials and methods

### Preparation and basic characterisation of G-CMC matrix for the 3D model

#### Chemical preparation of G-CMC

The pre-polymer components of the matrix (G-CMC), sodium carboxymethyl cellulose (CMC, 5 g), and bovine gelatin (G, 2.5 g) (Sigma-Aldrich, UK) were dissolved by adding gradually to 100 ml water while stirring. The mixture was heated to boiling, cooled to room temperature, and placed in the fridge overnight to ensure dissolution and then autoclaved at 121 °C for 25 min for complete polymerisation, microwaved, and held at 40 °C in a water bath. G-CMC was used at a 1:1 ratio with growth medium for all experiments.

#### G-CMC water absorption capacity

Swelling capacity was determined by freeze-drying batches of hydrated G-CMC (1 ml), whereby water was eliminated to dryness by sublimation under vacuum, overnight. This dried polymer was weighed and subtracted from the hydrated original for wet to dry weight ratios (w/w).

#### Viscoelastic properties of G-CMC vs CMC

A CVO 100 rheometer with smart software (Bohlin Instruments, Germany) was used to assess strain/stress (measure of mechanical strength) and viscosity in situ. G-CMC or CMC 1 ml aliquots were added to the cone/plate measuring system with a rotating upper cone and fixed lower plate and measurements were obtained in Pas = Pascal-second.

### Development of a 3D cancer spheroid model in G-CMC matrix

#### Determining optimum cell seeding and growth in G-CMC.

Cancer cells (colorectal HT29, breast MCF7; ACACC, Sigma-Aldrich) were dispersed into pre-warmed G-CMC gel:medium solution (Dulbecco’s Modified Eagle Medium with high glucose/L, GlutaMAX™-I, without phenol red, without HEPES and pyridoxine HCl (Sigma-Aldrich); supplemented with 10% foetal calf serum (FCS, First Link UK Ltd, Birmingham, UK) and 1% penicillin/streptomycin, 10,000 units and 10 mg per ml in 0.9% NaCl, respectively (P/S, GIBCO, Invitrogen, Paisley, UK)) in 24-well ultra-low binding plates, to achieve final concentrations of 25 × 10^3^, 50 × 10^3^, 100 × 10^3^, and 150 × 10^3^ cells/ml. The plates were cooled at 4 °C for 5 min to initiate gelling and then incubated at 37 °C, 5%CO_2_ in a humidified atmosphere. Cell growth was determined as equivalent to metabolic activity, at days 1, 4, 7, and 10. At each time, medium was replaced with 10% AlamarBlue™ Cell Viability Reagent (Thermo Fisher, UK) and cells incubated at 37 °C, ~ 5 h, to allow conversion of resazurin to resorufin, with resultant fluorescence intensity recorded (excitation/emission 530 nm/620 nm). Spheroid formation was confirmed by light microscopy and diameters evaluated by ImageJ (two optical fields per well). 100 × 10^3^ cells/ml was chosen for subsequent work.

#### Harvesting spheroids

For specific experiments, day 10 spheroids grown in G-CMC matrix were gently aspirated into 1-ml Eppendorf tubes, topped with sterile PBS and isolated using low speed centrifugation, at 100 g for 5 min. Frequent checks confirmed their sizes were equivalent to embedded spheroids (light microscopy).

#### Live/dead detection

Fluorescein diacetate and Propidium iodide were kept as stocks (FDA, 5 mg/ml acetone, − 20 °C; PI, 2.0 mg/ml phosphate-buffered saline, PBS, 4 °C; Sigma-Aldrich). FDA is catabolised by live cells giving green fluorescence and PI intercalates with dead cell DNA giving red fluorescence. For experiments, 8 μl FDA and 50 μl PI from stock were added to 5 ml of serum free medium and kept in the dark at 4 °C. The solution (1 ml) was added to freshly harvested cancer spheroids and incubated at room temperature for 4 to 5 min, in the dark. Spheroids were centrifuged, see above, and placed in Phenol red free medium, before imaging under the fluorescent microscope (Inverted EVOS FL; filter sets for Texas Red and FITC; excitation/emission 490 nm/610 nm).

#### Hypoxia detection

Image-iT Hypoxia reagent (Thermo Fisher) was added to the 3D model (spheroids within G-CMC) at 5–10 μM and incubated for 15 to 30 min. The compound is non-fluorescent when cells are at normal oxygen levels and becomes fluorescent at oxygen levels < 5%. Fluorescent intensity results were calculated numerically, with all individual points normalised for control untreated wells, and presented as % increase. (Nikon Eclipse TE300, adapted with a Hamamatsu camera [ORCA-*R*^2^] to increase emission/detection range to include NIR).

### Manufacture of anti-calreticulin gold nanoclusters

Targeted gold nanoclusters (AuNCs) were manufactured in-house, as previously described [16]. Briefly, AuNCs were coated with MSA and re-suspended in PBS (1 mg AuNCs/MSA/1 ml PBS). The NCs was conjugated to the anti-CRT antibodies using EDC as an acylating agent as follows: 200 µl of the AuNC/MSA (1 mg/ml) was mixed with 200 µl EDC (1 mg/ml) in PBS for 30 min at room temperature to initiate active symmetrical anhydride. One hundred microliter of anti-CRT solution (5 mg/ml) was added to the activated mixture and agitated gently for 2 h at room temperature. To separate the reagent and unconjugated AuNC/MSA, membrane centrifugal columns (Centricon) with a cut-off of 100 kDa were loaded with the mixture and centrifuged at 5,000 g, with UV monitoring at 280 nm of the retained samples. Validation of the conjugation of the anti-CRT to the AuNCs was carried out as before via dot blots (not shown) and detected by chemiluminescence [17]. Unconjugated AuNCs were used as controls.

### Targeting of cancer spheroids using anti-calreticulin gold nanoclusters

MCF7 and HT29 cells express CRT in 2D culture, shown by the binding of anti-CRT-AuNCs, but not unconjugated AuNCs, to formaldehyde fixed cells [16]. Here, the ability of anti-CRT-AuNCs to target and bind cancer cells was tested in live spheroids, to ensure that specificity of targeting was maintained in 3D cultures. Day 10 spheroids were harvested from G-CMC and incubated in 100 μl of anti-CRT-AuNCs for 2 h, rinsed, placed in PBS, and imaged using a fluorescence microscope (BX63 Olympus fluorescence microscope with cellSens software). To enable nuclear uptake and imaging of DAPI, some spheroids were also fixed in 4% formaldehyde prior to imaging. NC fluorescence was detected at 800 nm in the NIR. Controls spheroids were exposed to unconjugated MSA-coated AuNCs.

### Statistical analysis

Apart from viscosity measurements (*n* = 2), three independent repeats were conducted per protocol, unless indicated differently in figure legends. Multi-well experiments had technical repeats (3–4 wells/point). Where appropriate, results were analysed using 1-way ANOVA plus Tukey’s post hoc analysis, or Kruskal–Wallis plus Dunn post hoc analysis, on GraphPad Prism. Significance was set at *p* < 0.05.

## Results

### Physicochemical properties of G-CMC

**Fig. 1:**
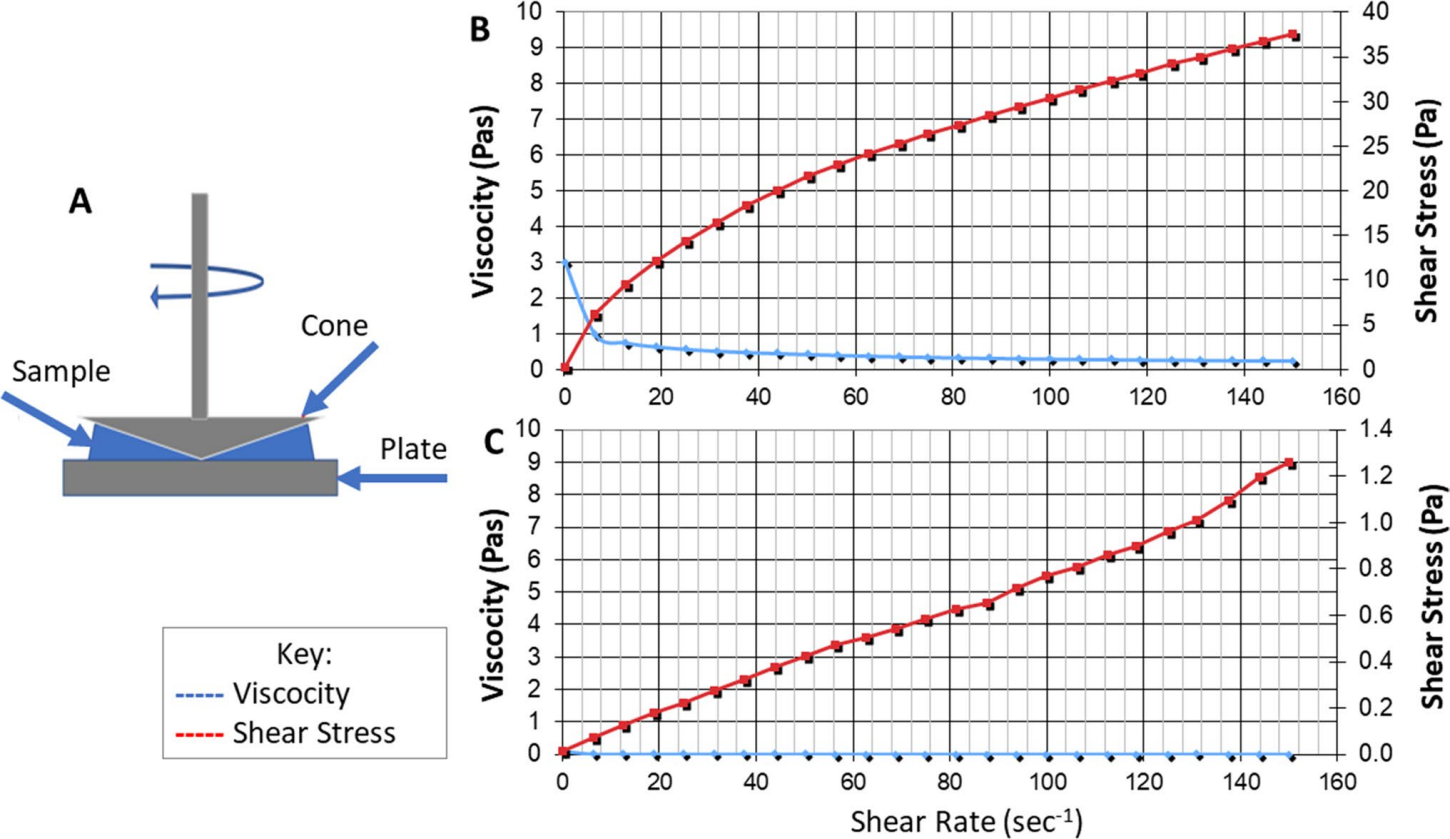
**a** The rheometer comprises a rotating upper cone and a fixed lower plate, with the mixture to be tested placed between the two. To measure viscosity, the upper cone is turned, and resistance recorded. Representative graphical representations of viscosity (blue line) and shear stress (red line) for **b** G-CMC and **c** CMC. Repeat experimental measurements were within 5% of these values

The mechanical strength and viscosity of G-CMC polymer were determined using a rheometer. Figure 1 shows that G-CMC exhibits a stronger mechanical strength with a recorded maximum of 38 Pas compared to CMC alone at 1.28 Pas. This makes it likely that G-CMC is strong enough for assembling and holding spheroids, while CMC by itself does not. Additionally, the viscosity is maintained throughout the stress sweep, with readings at 0.25 Pas for G-CMC versus 0.01 Pas for CMC alone. Too high a concentration would impede harvesting through centrifugation.

The water absorption/swelling capacity of G-CMC was determined by comparing wet to dry weights and was calculated as an average of 50 ± 3% (w/w ratios) as compared to CMC alone at 34 ± 2% (*n* = 3), indicating that the addition of gelatin resulted in higher hydrophilicity and higher cross-linking density of the polymer network.

### Growth of cancer spheroids in the 3D G-CMC matrix

**Fig. 2:**
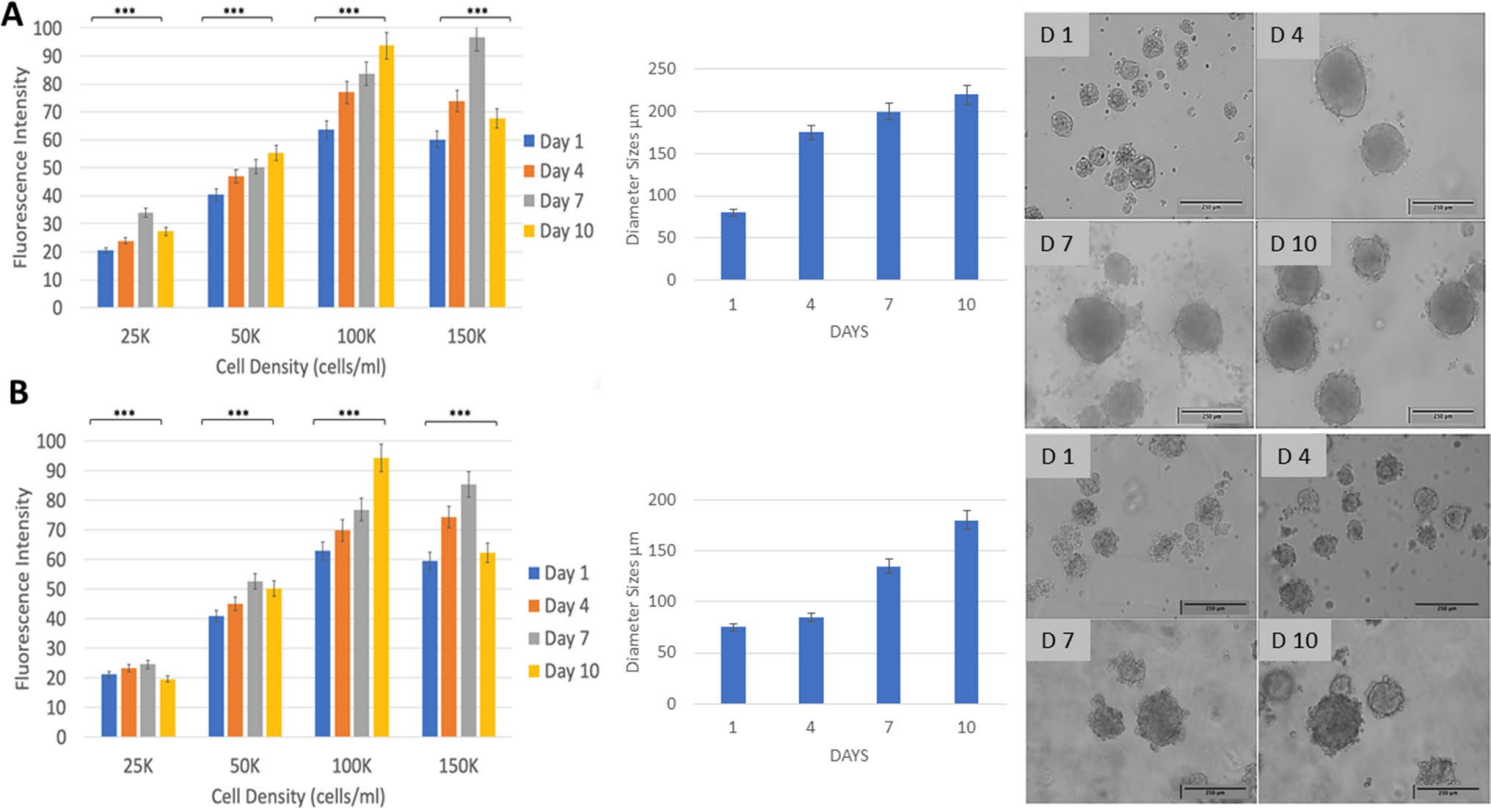
Growth of cancer spheroids in the 3D G-CMC matrix over 10 days. HT29 colorectal cells (**A**) and MCF7 breast cells (**B**) were seeded at a range of concentrations (25–150 × 10^3^cells/ml) for 1,4,7 and 10 days (*n* = 6). Growth is shown as equivalent to intensity of fluorescence (AlamarBlue™ assay; excitation/emission 530 nm/620 nm). Spheroids, for the 100 × 10^3^/ml cell seeding concentration, were imaged by light microscopy (representative images shown) and diameters calculated, ImageJ. One-way ANOVA, Tukey’s post hoc analysis (***p < 0.05)

A range of HT29 colorectal and MCF7 breast cancer cell seeding concentrations were evaluated for spheroid growth and formation in the G-CMC matrix over 10 days and quantified by metabolic activity, to reflect growth, and by microscopy for morphology and spheroid sizes (Fig. 2). For both cell lines, the lowest cell seeding (25 × 10^3^/ml) did not demonstrate an increase in growth over time, while the highest cell seeding (150 × 10^3^/ml) showed a marked decrease in growth between day 7 and 10 day by over 20%, suggesting over-confluence at these conditions.

Linear growth was demonstrated at cell seeding of 100 × 10^3^/ml. This concentration was used for all further experiments for both cell lines. Spheroid diameters measured at this concentration confirmed growth optically. Interestingly, for HT29 spheroids, sizes increased markedly from 1 to 4 days from 80 to 175 μm, with increases to 200 μm on day 7 and 220 μm on day 10. Microscopic images confirmed that spheroid sizes were increasing, appearing dense and compact over time. Similarly, MCF7 breast cancer spheroids were also forming by day 1 with average sizes of 75 μm, continued with averages of 85 μm on day 4, 135 μm on day 7, and 180 μm on day 10. Generally, breast cancer spheroids were smaller and appeared denser than colorectal cancer spheroids.

**Fig. 3:**
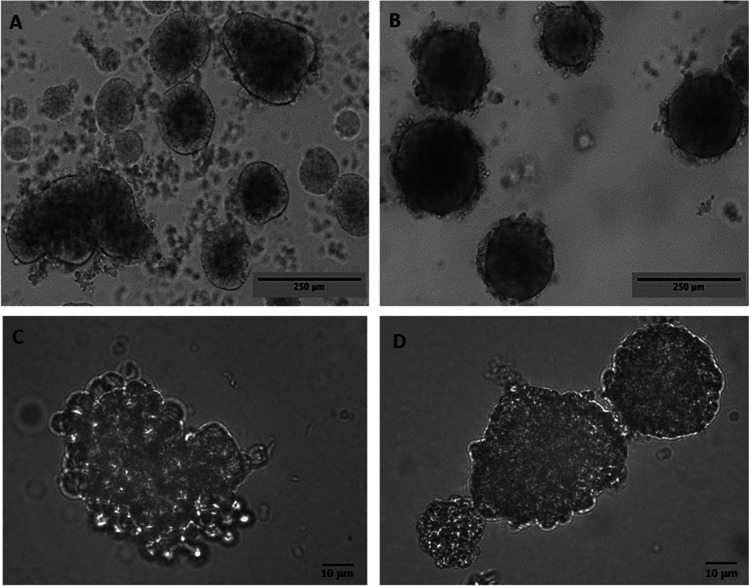
HT29 3D spheroids (**A**, **C**) and MCF7 3D spheroids (**B**, **D**) grown in 3D G-CMC matrix for 10 days, harvested by centrifugation (100 g, 5 min) and embedded into fresh G-CMC. Representative images, showing some debris in HT29 cultures. Magnification bars = 250 μm, **A**, **B**; 10 μm, **C**, **D**

G-CMC 3D cultures grown for 10 days were centrifuged at 100 g to determine whether we could isolate intact spheroids, and these were placed into fresh G-CMC for measurements. Generally, spheroids did not disintegrate but retained their sizes **(**Fig. 3), comparable to those that were observed while still matrix-embedded (≅ 200 μm, shown Fig. 2). Small cellular debris were sometimes observed in HT29 cultures, suggesting that the cells in the spheroids were held together more loosely than those in MCF7 spheroids. Further experiments were carried out either in matrix-embedded or harvested spheroids.

**Fig. 4:**
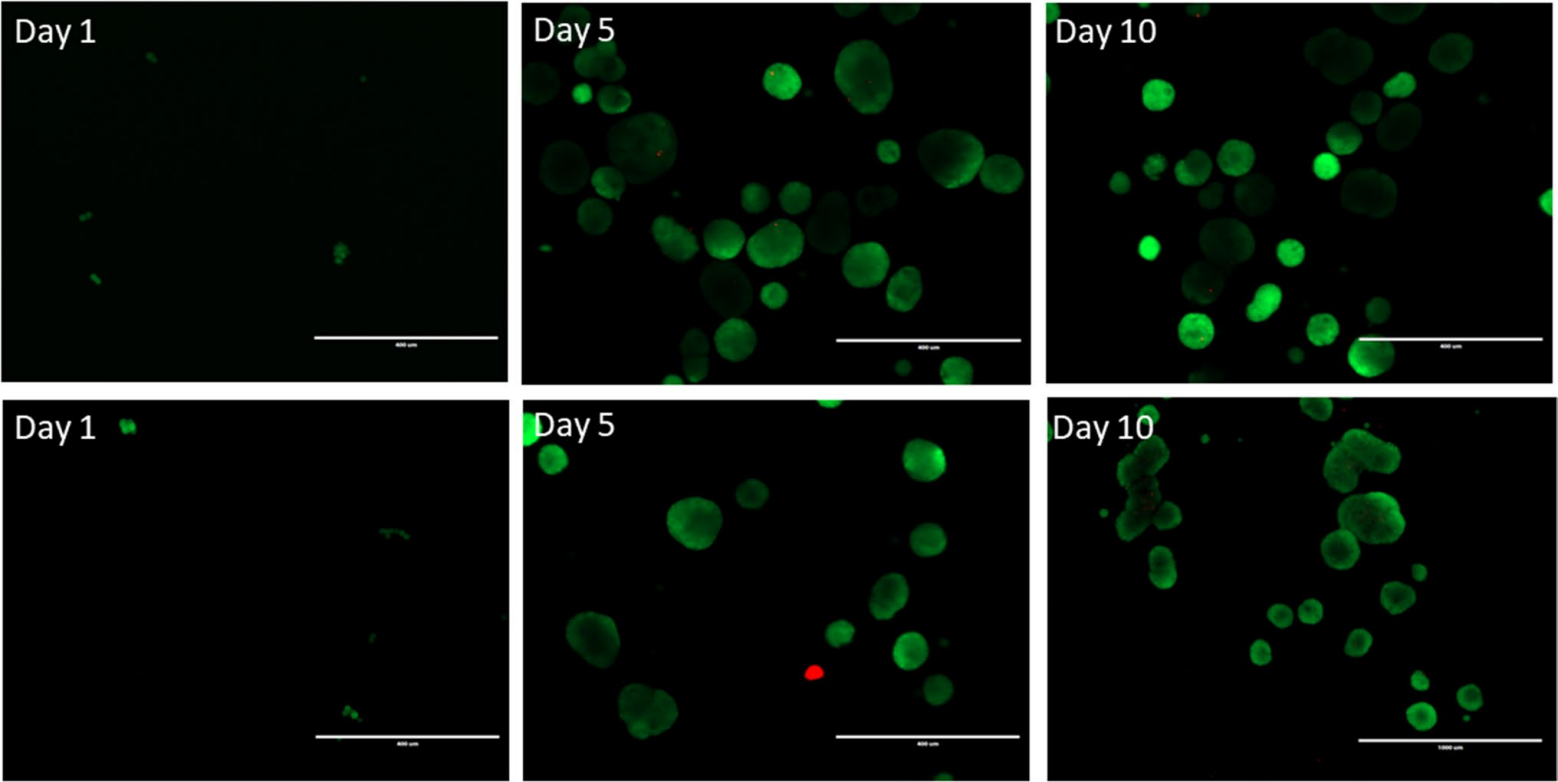
Live/dead assay in HT29 3D spheroids (top panel) and MCF7 3D spheroids (bottom panel) grown in 3D G-CMC matrix, over time (representative images). Spheroids were harvested and exposed to fluorescein diacetate (FDA) and propidium iodide (PI), which stain viable cells (green) and dead cells (red). Fluorescent signal is weak in day 1 cultures due to small numbers, for both cell lines. Magnification bar = 200 μm

Viability for both colorectal and breast cancer spheroids grown in G-CMC 3D matrices was confirmed by using the live/dead FDA/PI assay on spheroids harvested at three time points. Fluorescent images show scarce cell death (red), with the vast majority exhibiting FDA (live indicator) uptake (green) (Fig. 4).

Hypoxia was determined by incubating G-CMC embedded spheroids with the Image-iT Hypoxia reagent, which fluoresces at < 5% oxygen. Fluorescence intensity measurements, normalised against individual controls, showed minimal hypoxia at day 1, with levels increasing to ~ 100% by day 10 (days 1, 4, 7, and 10: HT29; 0 ± 5%, 32 ± 8%, 81 ± 10%, 95 ± 12%; MCF7; 14 ± 6%, 20 ± 12%, 72 ± 8%, 104 ± 24%; *p* < 0.05, day 4 v day 1, Kruskal–Wallis).

### Targeting of cancer spheroids using anti-calreticulin gold nanoclusters

**Fig. 5:**
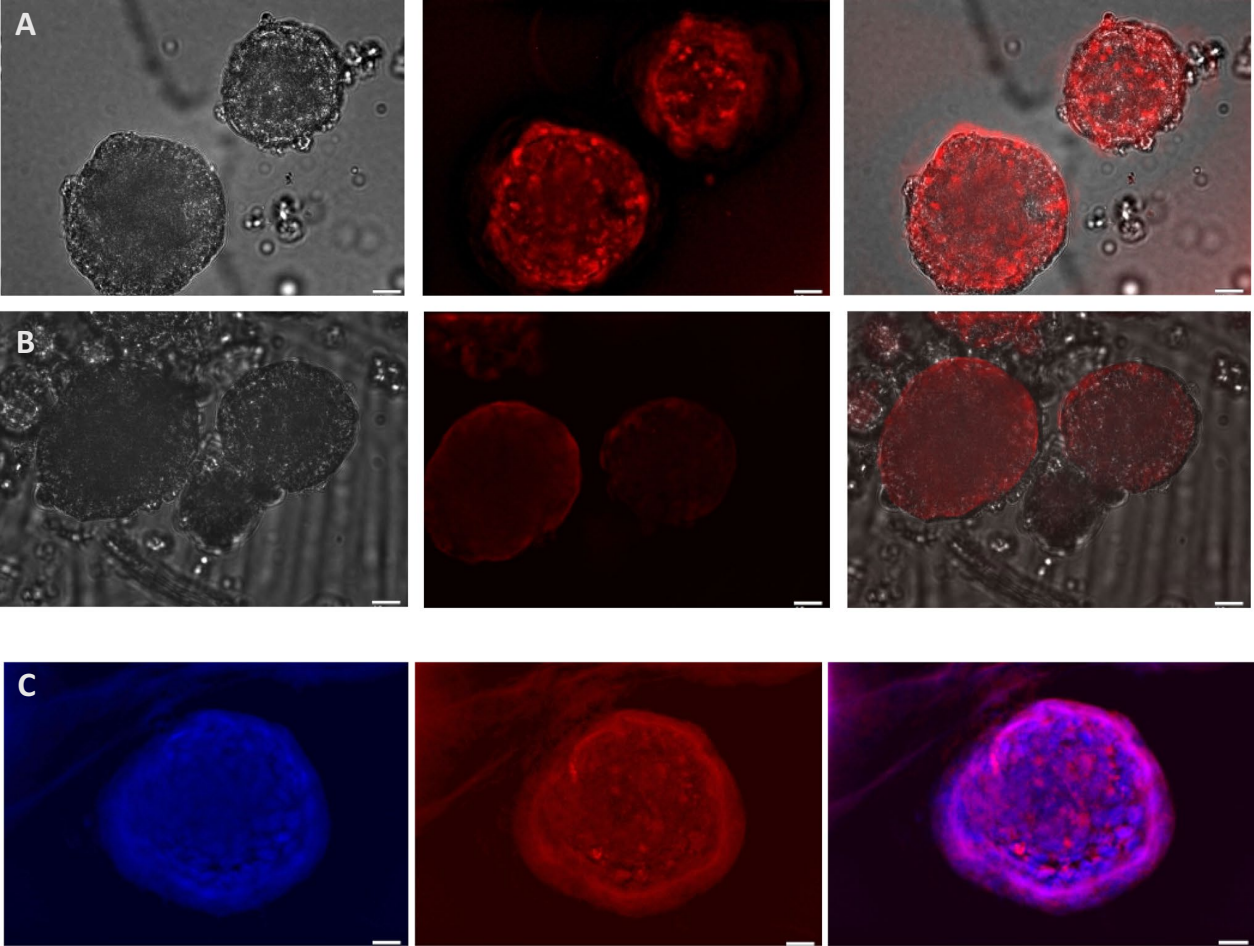
Expression of calreticulin by HT29 spheroids grown in G-CMC matrix for 10 days (representative images). Spheroids were harvested, incubated with anti-CRT-AuNCs for 2 h and imaged live, using fluorescent microscopy. **A** Uptake of anti-CRT-AuNCs. **B** Uptake of un-targeted, unconjugated control AuNCs. Both **A** and **B** series of images (L-R): light, fluorescence; merged; with NIR red pseudocolour. **C** Spheroids exposed to anti-CRT-AuNCs (red) were fixed prior to imaging, to allow for staining with nuclear DAPI (blue); L-R: DAPI fluorescence, NC fluorescence; merged. Bar = 10 μm; magnification × 60, immersed objective, Olympus BX63)

**Fig. 6:**
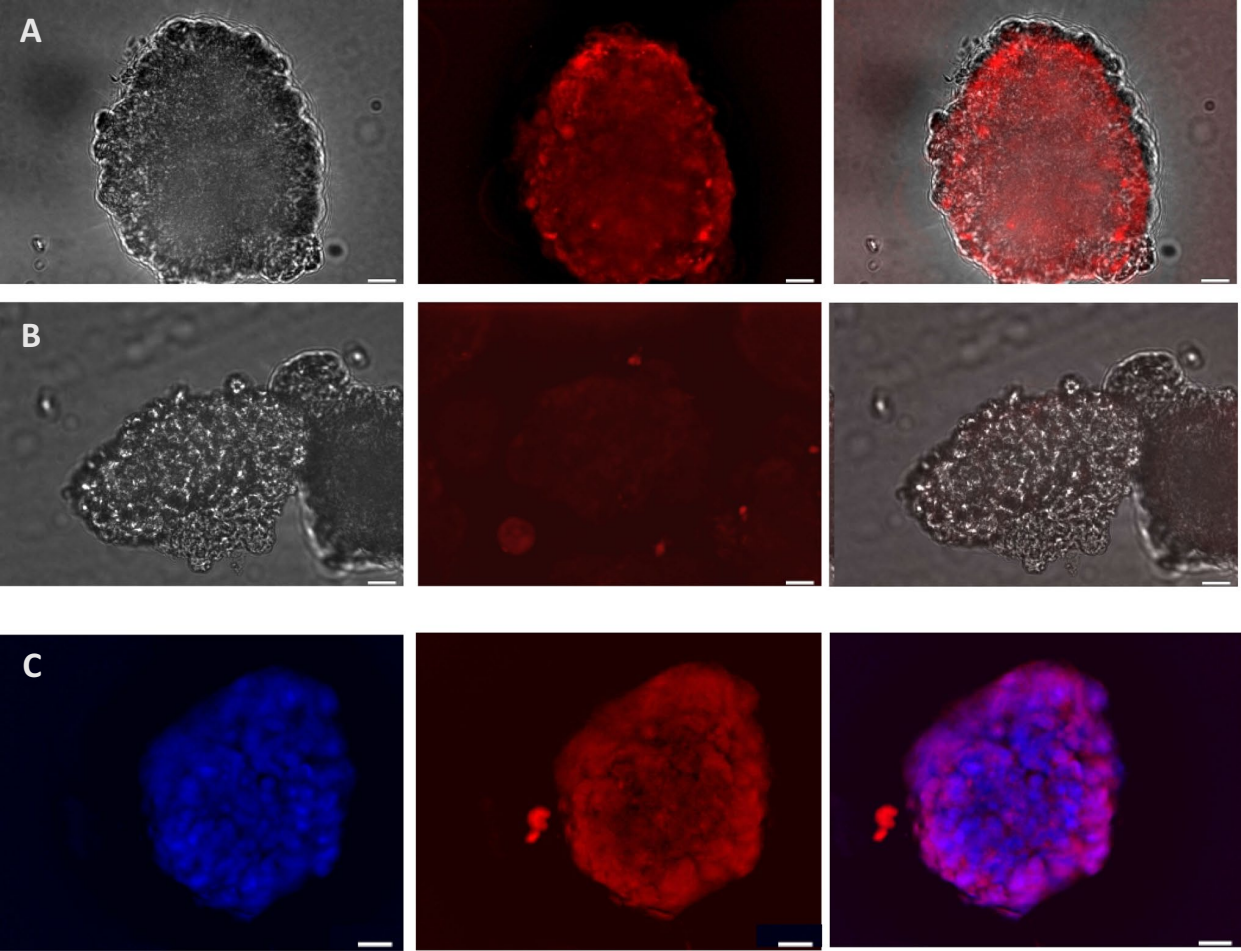
Expression of calreticulin by MCF7 spheroids grown in G-CMC matrix for 10 days (representative images). Spheroids were harvested, incubated with anti-CRT-AuNCs for 2 h and imaged live, using fluorescent microscopy. **A** Uptake of anti-CRT-AuNCs. **B** Uptake of un-targeted, unconjugated control AuNCs. Both **A** and **B** series of images (L-R): light, fluorescence; merged; with NIR red pseudocolour. **C** Spheroids exposed to anti-CRT-AuNCs (red) were fixed prior to imaging, to allow for staining with nuclear DAPI (blue); L-R: DAPI fluorescence, NC fluorescence; merged. Bar = 10 μm; magnification × 60, immersed objective, Olympus BX63)

Monolayer cultures of HT29 and MCF7 cancer cells were previously shown to express CRT, using anti-CRT-AuNCs [16]. We tested whether live, harvested cancer spheroids, maintained CRT expression, by incubating with anti-CRT-AuNCs, for 2 h. We imaged the live spheroids under fluorescence and showed that spheroids from both cancer cell lines bound anti-CTR-AuNCs strongly, with minimal passive uptake of non-targeted, unconjugated control AuNCs. Additional detection of cell nuclei was carried out in spheroids which were fixed prior to imaging, as fixation allows for the penetration of the DAPI nuclear stain (Figs. 5 and 6).

## Discussion

3D in vitro culture is believed to be more representative of the native tissue than 2D culture, based on both morphology and cell behaviour [1, 4, 5, 18, 19]. Importantly, response to drugs is often different between 3 and 2D cultures, especially in cancer models, which poses a significant challenge to drug discovery and translation to the clinic. We developed a versatile model of breast and colorectal cancer to address specific difficulties associated with 3D cellular growth in in vitro matrices. Our primary objective was to be able to remove the cancer spheroids intact out of the 3D matrix with minimum disruption and transfer into matrix free environment for imaging and/or exposure to new anticancer drugs. We recognise that this step would also enable further precise interrogation of the cells. We also wanted to demonstrate targeting of the cancer cells using imageable nanoparticles, in live culture.

Hydrogels of various compositions have been proposed for 3D culture [20]. However, isolation of cancer spheroids from current models is problematic due to disintegration of the spheroids during the process. We therefore evaluated the efficacy of gelatine-carboxymethyl cellulose (G-CMC) hydrogels to support growth of cancer spheroids. We chose to explore a matrix of CMC and gelatin, as combinations of the two results in hydrogels which have rapid hydration, swelling and diffusion properties, and ease of manufacture. Gelatin-containing matrices tend to have a porous nature and have been described as biocompatible. This is due to the presence of gelatin, the hydrolysed product of collagen type 1, the most abundant extracellular matrix protein, which retains domains that bind to cell-surface receptors and to extracellular matrix proteins [11]. This provides the right environment for the attachment of adherent cells, such as colorectal and breast carcinomas [20, 21]. Crossed-linked gelatin hydrogels have been shown to support the adhesion, proliferation, and differentiation of adipose tissue-derived stromal cells (ADSCs) [22]. Furthermore, a highly cross-linked highly G-CMC hydrogel has been used for initiation of vascular networks in 3D [14].

A wide range of methods are currently explored to define biophysical parameters, from atomic force microscopy to rheology and modelling. We chose to define the biophysical properties of the novel G-CMC mixture by measuring shear stress (as an indication of mechanical strength) and viscosity, using rheology, and water swelling capability. The water swelling capability was determined via freeze–thaw cycles and the addition of gelatin to CMC increased w/w ratios from 36% for CMC alone to 50% for G-CMC. This is an indication of the cross-linking density of the polymer, with stiffer constructs showing less capacity for swelling, and also, in this case, an indication of higher hydrophilicity, as described previously [20]. The addition of gelatin to CMC (G-CMC) also resulted in a stronger mechanical strength with a recorded maximum of 38 Pas compared to 1.28 Pas for CMC alone. Additionally, the viscosity was maintained throughout the stress sweep. We hypothesized that this makes it likely that G-CMC is strong enough for assembling and holding spheroids, while CMC by itself does not, as our G-CMC results were of the order of magnitude reported for cell-growth supporting matrices, such as Matrigel (180 Pa) and collagen hydrogels, from 65 Pa (in-house measurements for 0.2% collagen hydrogels) to 330 Pa [23].

The cancer spheroids generated within G-CMC were similar in their timeline and growth trajectory to those generated in other matrices containing collagen, gelatin, or Matrigel [24] reported by various groups. In our study, for both colorectal (HT29) and breast (MCF-7) cancer cells, we observed small spheroids form from day 1, increasing in size up to day 7, with growth largely stabilising (days 7–10), with spheroids typically at 200 μm and 180 μm diameter for HT29 and MCF7, respectively. There was excellent viability throughout, regardless of increasing hypoxia levels. Reasons for this growth timeline have been discussed extensively in the literature and include hypoxia (with a diameter of ~ 400 μm the critical point at which hypoxia becomes catastrophic and necrosis starts), nutrition penetration, and limited metabolic waste transport, both within a cell spheroid, but also through a matrix structure [25, 26].

At the final end-point, day 10, we centrifuged the 3D mixture at 100 g for 5 min and harvested the cancer spheroids generally intact. To our knowledge, few successful spheroid isolation methods from a 3D matrix have been reported, for example, the separation of gastric cancer spheroids from alginate-gelatin-Matrigel hydrogel [24], while other approaches require to liquefy the matrix itself. We consider the isolation of whole spheroids an advantage, as spheroids can be transferred between various culture conditions, but also—after experimentation—they can be removed from the G-CMC matrix for further interrogation, either by imaging, as we demonstrate here, or for further cellular and molecular investigations.

Having defined the parameters of colorectal and breast cancer growth within G-CMC, we aimed to demonstrate specific imaging of cancer spheroids, with biomarker targeted nanoparticles. We used our previously developed gold-based nanoclusters (AuNCs) which emit in the near infrared region, NIR [16]. These are typically very small (10 nm), with a long half-life (> 3 years; laboratory data). We conjugated AuNCs to a peptide we synthesized which binds to a short sequence of the calreticulin protein. Validation of the conjugation between the AuNCs and the anti-CRT antibody was carried out via dot blots, using increasing concentrations of “cold” antibody (not shown). Calreticulin (CRT), a cytoplasmic calcium-binding protein, is predominantly found in the endoplasmic reticulum. The protein is multifunctional and appears to act as control chaperone by stopping the proteins that are misfolded from progressing to the Golgi apparatus. CRT also acts as Ca^2+^ storage protein, therefore playing an essential role in the intracellular signal transduction systems, influencing tasks such as cellular proliferation and death. It also acts as a nuclear hormone receptor gene transcription modulator. The targeted nanoclusters, anti-CRT-AuNCs, were previously shown to bind to HT29 and MCF7 cancer cells lines, grown in 2D monolayer cultures, demonstrating the potential of calreticulin as a cancer biomarker [16, 27]. The results indicated that CRT exists on the surface of cancer cells suggesting that CRT translocates to the cell surface during carcinogenesis.

To determine whether anti-CRT-AuNCs could be used as fluorescent bio-probes which bind to cancer cell surface-bound CRT, we tested their binding in live, 3D cancer spheroids. As expected, live spheroids bound anti-CRT-AuNCs demonstrating that calreticulin is conserved on the cell membrane, similar to our previous report [16]. Some minimal uptake of unconjugated control AuNCs was also observed, a non-specific process which is dependent on size and charge [28]. We recognise that a thorough exploration of time-dependent uptake and release of both targeted and untargeted NCs would be very informative, with reports showing deeper penetration of gold nanoparticles into spheroids over longer time periods of incubation [29]. This would be the subject of a distinct study which would aim to translate to in vivo models.

Overall, the use of 3D models is more representative of in situ conditions and essential for the evaluation of nanoparticles as the latter have been shown to have different behaviour in the presence of the tumour 3D geometry and microenvironment. This has been demonstrated in a number of studies, e.g., [30–32] in an alginate-based 3D versus 2D model of prostate cancer where targeted nanoparticle mediated gene delivery was only observed in 3D. Our study, which describes a novel 3D in vitro model of cancer which allows versatility in the handling and interrogation of cancer spheroids and demonstrates nanocluster-mediated targeting of cancer, is a valuable addition to the field of 3D models which recapitulate in vivo scenaria.
